# IFN-*α* Boosting of* Mycobacterium bovis Bacillus* Calmette Güerin-Vaccine Promoted Th1 Type Cellular Response and Protection against* M. tuberculosis *Infection

**DOI:** 10.1155/2017/8796760

**Published:** 2017-09-27

**Authors:** C. E. Rivas-Santiago, G. G. Guerrero

**Affiliations:** ^1^Unidad Académica de Ciencias Biológicas, Laboratorio de Bioquímica Molecular e Inmunobiologia, Universidad Autónoma de Zacatecas, Av. Preparatoria S/N. Col. Agronómicas, 98066 Zacatecas, ZAC, Mexico; ^2^Catedra-CONACYT, Avenida Insurgentes Sur 1685, Guadalupe Inn, 01020 Álvaro Obregón, CDMX, Mexico

## Abstract

The role of type I IFNs in the pathogenesis and control of mycobacterial infection is still controversial. It has been reported that type I IFNs exacerbated* M. tuberculosis* infection through hampering Th1 type cellular immune response. However, under certain conditions they can act as natural immune adjuvants for commercial vaccines. At this point, we have reported recently that successive IFN-alpha boosting of* Mycobacterium bovis Bacillus Calmette Güerin* (BCG) vaccinated mice protected adult mice from intradermal* M. lepraemurium* infection and a difference in iNOS was observed. In the present work, we have found that intramuscular IFN-*α* boosting of* Mycobacterium bovis Bacillus Calmette Güerin* (BCG) vaccine, either in vitro (human cell line or macrophages derived from PBMC) or in vivo (aerosol mouse model of* MTb* infection), promoted mostly the development of specific anti-antimycobacterial Th1 type cytokines (IFN-*γ*; IL-12, TNF-alpha, and IL-17; IL1*β*) while bacterial load reduction (0.9 logs versus PBS or BCG vaccine) was observed. These findings indicate that, under the experimental settings reported here, interferon alpha can drive or affect the TH cellular immune response in favour of BCG-inducing immunity against* M. tuberculosis* infection.

## 1. Introduction

Th1 type cellular activity plays a key role for the control of* M. tuberculosis (MTb)* as well as the induction of long-term memory conferring protective immunity [1–4]. To this end heterologous prime boost strategies have been shown as a potent and effective alternative to promote not only immunological memory but they also can trigger innate immune responses independent of T cell immunity [5, 6]. Therefore, in order to better understand* MTb*-specific immune mechanisms, both cellular and innate immune responses should be taken into account and the role of pivotal cytokines orchestrating their complex interactions should be elucidated [7]. One important family of infection-induced cytokines is type I interferons (IFN-I) (*α*/*β*). IFN-I promote differentiation/activation of DCs in both human and mice and play an important role in long-term survival of CD8+T cells in response to specific antigens (Ag) [8, 9], and importantly in the regulation of the Th1 responses [8]. The role and influence of the type I IFNs as mucosal adjuvants against influenza virus and in cancer immunotherapy is well documented, on innate [10–13] and adaptive cell populations [14, 15]. However, contrasting and controversial results have been provided against microbial infections in vivo as well as in vitro [16]. Thus, mice infected with* MTb* hypervirulent clinical isolate HN878, and treated intranasally with IFN-*α*, were unable to restrain bacterial growth, presumably due to a failure to develop Th1 type immunity [17]. A similar negative effect has been observed in patients infected with hepatitis C virus and* M. tuberculosis*; IFN-alpha monotherapy exacerbated the disease [18]. Two recent studies pinpoint the potential role of type I IFNs in antimycobacterial infections. First, an inducible type I IFNs transcriptional signature in white blood leucocytes (neutrophils) correlated with active tuberculosis determined by microarrays and flow cytometry [19]. Second, targeting host innate inflammatory response can mediate IL-1*β* eicosanoid production that limits excessive production type I IFNs, leading to a host resistance to* M. tuberculosis* infections. Therefore, this crosstalk represents a potential target against multidrug resistant* MTb *strains and/or chemotherapy in human tuberculosis [20]. At this point, our group have reported that IFN-alpha associated with BCG protects against* M. lepraemurium*; a mice pathogen elicited a similar skin lesion to* M. leprae* in adults persons with a concomitant increase in NO synthase [21]. On the other hand, it has been reported that IFN-beta can augment BCG immunogenicity and IL-12 production [22]. BCG vaccine still showed a high variability and loss of prolonged cellular and immunological memory during adulthood [23–25]. Under this scheme of type I IFNs, in this work we sought to investigate whether IFN-alpha boosting of BCG vaccine can drive Th1 inducing capacity and protect against* M. tuberculosis* infection.

## 2. Materials and Methods

### 2.1. Animals

Pathogen-free BALB/c mice (seven- to eight-week-old) were obtained from Harlan, Co., France, and were maintained in a specific pathogen-free environment of the Institut Pasteur, campus Lille (Lille, France) throughout the whole experiment. All animal experiments were performed and approved by the Institutional Animal Care and Use Committee, and vaccination/infection experiments were made in the biosafety facilities level 2 (BSL2) and 3 (BSL3).

### 2.2. Microorganisms

The BCG Pasteur strain (isolate 1173P2, World Health Organization, Stockholm, Sweden) was grown as dispersed cultures in Sauton medium for 14 days as described [26] and stored at –80°C until use. The* M. tuberculosis* H37Rv strain used for the mice infection was also grown as reported [26]. For the human infections assays,* Mycobacterium tuberculosis* H37Rv strain (ATCC 27294) was obtained from National Institute of Respiratory Diseases (NIRD) MEXICO, and* Mycobacterium bovis* BCG (ATCC 27291) was obtained from National Institute of Medical Sciences Nutrition (NINMCSZ). Logarithmically growing cultures were centrifuged at 800 rpm for 10 min to eliminate clumped mycobacteria and then washed three times in RPMI 1640. Mycobacteria were resuspended in RPMI 1640 containing 10% FCS and then stored at −80°C.

### 2.3. Immunizations

Groups of BALB/c mice seven to eight weeks old were immunized subcutaneously (s.c.) with 5 × 105 colony-forming units (CFU) BCG in 50 *μ*l sterile phosphate-buffered saline (PBS) or with 50 *μ*l sterile PBS. At intervals of two weeks during a total of six weeks, each group of mice was boosted intramuscularly (i.m.) with 100 *μ*l PBS or IFN-I-*α* (100 UI). On the other hand, for intranasal immunizations, mice were anesthetized and instilled into nostrils with 20 *μ*l of aPBS solution containing Ag85B (5 *μ*g/ml) once. Vaccination was performed four days after the last IFN-*α* boost. Fourteen days after antigen administration, mice were sacrificed, and organs were obtained.

### 2.4. Mice* M. tuberculosis* Infection

Aerosol* M. tuberculosis (MTb)* infections were performed as described [26]. Mice were first anesthetized by intraperitoneal (i.p.) route with a dose of pentobarbital sodium equivalent to a 1/10 of the mouse body weight, followed by aerosol challenged using a homemade nebulizer and an inhaled dose of 100–150 CFUs. Eight weeks after infection, viable bacteria numbers were measured by plating serial dilutions of whole organ (spleen or lung) homogenates on Middlebrook 7H11 solid medium supplemented with 2 mg/ml of THF(2-thiophenecarboxylic acid hydrazide). Plates were incubated at 37°C/5% CO2 for 21 to 25 days. Colonies were counted, and total CFU/organs were calculated and normalized.

### 2.5. Macrophages Derived from PBMC

Peripheral whole heparinized blood was collected by venipuncture from healthy men at University Autonomous of Zacatecas. PBMC were isolated from whole heparinized venous blood by Ficoll gradient centrifugation. Briefly, whole blood was diluted 1 : 1 with l-glutamine supplemented RPMI 1640 medium and subjected to gradient density centrifugation (1200 rpm, 45 min, 21°C) over Ficoll-Paque. Following removal from the interface, PBMC were washed three times in RPMI 1640, resuspended in complete culture medium, counted, and adjusted at required concentrations. Viability of PBMC was 98–100% by trypan blue exclusion in all experiments. PBMC (5 × 10^6^) were plated in 6-well plates (BD Falcon, Franklin Lakes, NJ) in 3 mL of complete culture medium per well and incubated at 37°C in a humidified 5% CO_2_ environment for two hours. Following incubation, nonadherent cells were removed by washing twice with RPMI 1640 supplemented with Penicillin/Streptomycin/Glutamine (P/S/G). Plastic adherent cells (monocytes) were cultured at 37°C in humidified 5% CO_2_ environment for 7 days, which allows for differentiation into macrophages (MDM).

### 2.6. Human Cell Infection

A549 cell monolayer (150,000 cells) or derived macrophages from PBMC were put in 96/6-well plates, incubated with 5% CO_2_/37°C for 24 h. Thereafter, cell monolayers were washed to eliminate nonadherent cells and were treated as follows during 24 h: (1) cells without stimulation; (2) cells stimulated with BCG suspension with a multiplicity of infection (MOI) of 0.1 : 3.3; (3) cells stimulated with 500 UI/ml of IFN-*α*; and (4) cells stimulated with BCG (MOI 0.1 to 500 UI/ml) of IFN-*α*. Afterwards, each group of cells were washed with Hank's solution (HBSS) (Gibco, Co) and infected with a MOI of 10 : 1 (bacteria : cell ratio). Four hours after infection, cell monolayer was washed gently (three times) with HBSS and incubated with 60 *μ*g/ml of amikacin/F12 fresh medium (Gibco) to 37°C. Cells were resuspended in complete medium and cultured for the times indicated in each experiment. At each time-point, cells were lysed with SDS at 0.25% and 500 *μ*l BSA at 4%. Serial dilutions of the bacterial suspensions were plated (in duplicate) on supplemented Middlebrook 7H11 solid medium with 2 mg/ml of THF (2-thiophenecarboxylic acid hydrazide). Petri dishes were kept in sealed plastic bags at 37°C for 3-4 weeks.

### 2.7. Cytokine Determination

For cytokine measurements single suspensions of lymph node, spleen or lung cells were prepared as described elsewhere. Briefly, spleen, or lymph nodes were crushed gently in a Falcon sieve using the top end of a 1 m l syringe piston. The excised lung tissue was minced and incubated for 1 h at 37°C in PBS containing 2% fetal bovine serum (FBS), 125 U/ml of Collagenase I (Sigma Aldrich). Single cells suspension of spleen, lymph node, or lung cells were prepared and used for in vitro culture. 1 × 10^5^ a 1 × 10^6^ viable cells were cultured in RPMI 1640 medium (GIBCO, BRL) supplemented with 10% heat-inactivated FBS, 2 mM L-glutamine, 10 mL HEPES, 100 U/ml of penicilin G, 100 *μ*g/ml de Streptomycin, and 0.05 mM 2-mercaptoethanol. Cells were incubated in 96-well ELISA plates with Ag85B (2.5 *μ*g/ml) or HBHA (2.5 *μ*g/ml). Cells were incubated at 37°C in an atmosphere of 5% CO_2_ for 72 h. After this incubation time, cell culture supernatants were collected and stored at −80°C until analysis. Amounts of IFN-*γ*, TNF-*α*, and IL-12; IL1*β* and IL-17; IL-10 in the supernatants were measured by using a specific sandwich ELISA (OptEIA; BD Bioscience Pharmingen) according to the manufacturer's instructions. Data are expressed as the mean ± SEM for each mouse group.

### 2.8. Statistical Analysis

Statistically significant differences among groups were determined using One-way ANOVA, with Prism 5.0 software (Graph Pad Software, Inc. San Diego, Ca). A difference between the means was calculated by Tukey's test. *P* < 0.05 was considered significant.

## 3. Results

### 3.1. Specific Th1 Type Cytokine Production after IFN-*α* Boosting of BCG-Vaccinated Mice

**Figure 1:**
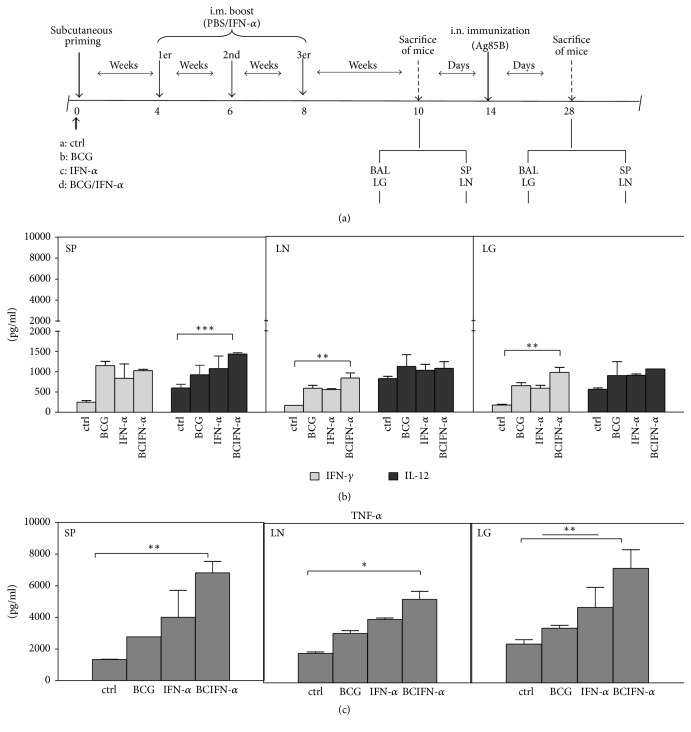
(a) Experimental schema of the prime boost protocol designed for the study. Adult BALB/c mice were s.c. primed with PBS or 5 × 10^5^ CFUs BCG. 4, 6, and 8 weeks later, mice of each group received PBS or 100 UI IFN-*α* i.m. Two weeks after i.n. with Ag85B, spleen, lymph node, or lung cells from PBS control mice, BCG-vaccinated mice, IFN-alpha, or BCG-vaccinated/IFN-alpha boosted mice were cultured in the presence of medium (RPMI1640/2% FBS) only or Ag85B (2.5 *μ*g/ml). (b-c) Levels of cytokines were measured after 72 h culture in the supernatants by using the OptEIA™ kit (BD Biosciences). Values are expressed in pg/ml and represent media ± SEM of samples tested in duplicate from each group of mice. Differences are significant at *P* < 0.05 with respect to control PBS immunized mice or to BCG-vaccinated without boost (*∗*, *∗∗* or *∗∗∗*).

**Figure 2:**
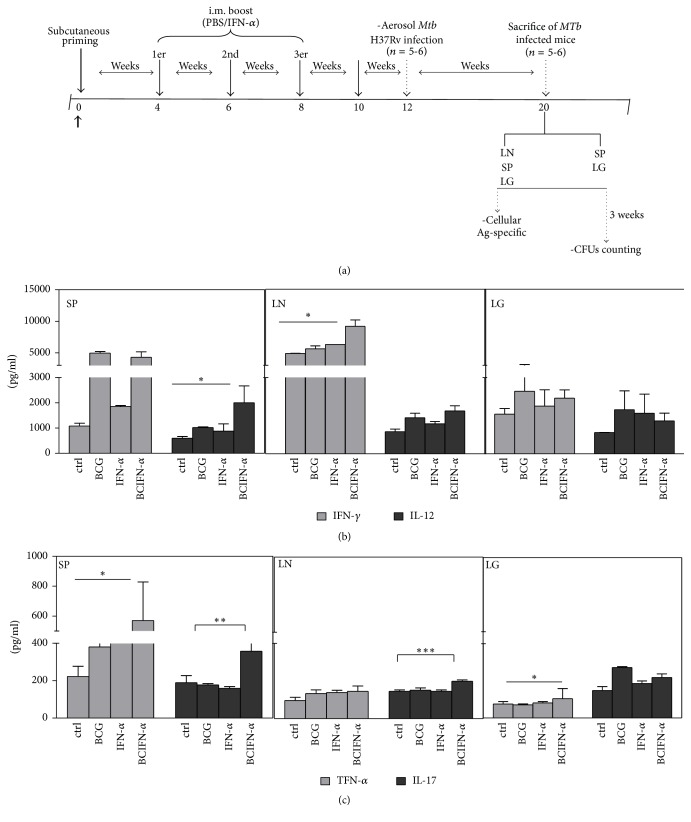
(a) Schematic representation of the designed protocol used in this study. Seven- to eight-week-old adult BALB/c mice were primed with PBS (group a); 100 UI of IFN-*α* (group b); 5 × 10^5^ CFUs of BCG Pasteur (group c); and 5 × 10^5^ CFUs of BCG + 100 UI of IFN-*α* (group d). Four, six, and eight weeks after the priming, mice from groups a and c received PBS, while mice from groups b and d received IFN-*α* (100 UI), respectively. Two weeks after the third boost, mice were infected by aerosol route with a low dose (100 CFUs) of* M. tuberculosis* H37 Rv. Eight weeks after infection, mice were sacrificed to determine CFU counts in the lungs and spleen. (b-c) Cell culture of lymph node, spleen, and lung was stimulated in vitro with HBHA and cytokine production was measured by ELISA. Values are expressed in pg/ml and represent media ± SEM of samples tested in duplicate from each group of mice. Differences are significant at *P* < 0.05 with respect to control PBS immunized mice (*∗*) and with respect to BCG vaccination without boost (*∗∗*).

It has been shown that type I IFNs can hamper Th1 inducing capacity, leading to an exacerbation of the* MTb* infection [17]. In this work, first, we investigated whether IFN-alpha boosting on BCG-Th1 inducing capacity was able to promote specific Th1-type cytokines. Thus, by using a prime-boost protocol (Figure 1(a)), we have found that splenocytes from BCG-vaccinated/i.m. and IFN-*α* boosted mice, followed by i.n. immunization with Ag85B, did not produce IFN-*γ* (1025 ± 36 pg/ml) production compared to BCG-vaccinated mice (1150 ± 107 pg/ml) (*P* < 0.05) (Figure 1(b) gray bars); it elicited a higher amount of IL-12 (1436 ± 37 pg/ml) (Figure 1(b), dark gray). In contrast, lymph node from BCG-vaccinated/i.m. IFN-alpha boosted mice i.n. with Ag85B produced a slight increase of IFN-*γ* (840 ± 125 pg/ml) (*P* < 0.05) (Figure 1(b), gray bars), compared with BCG-vaccinated (587 ± 72 pg/ml) (*P* < 0.05) (Figure 1(b)), IFN-*α* (556 ± 18 pg/ml) and PBS immunized mice (166 ± 0 pg/ml) (*P* < 0.05) (Figure 1(b)). No increase of IL-12 production was observed (Figure 1(b), dark gray). On the other hand, lung cells from lung cells from BCG-vaccinated/i.m. IFN-*α* boosted mice (Figure 1(b), gray bars) elicited a moderate increase of IFN-*γ* (975 ± 125 pg/ml), significantly compared to nonvaccinated control mice (IFN-*γ*, 175 ± 18 pg/ml)) (*P* < 0.05) and BCG-vaccinated mice, nonboosted mice (IFN-*γ*, 650 ± 72 pg/ml;) (*P* < 0.05) (Figure 2(c), gray bars). Although BCG-primed/i.m IFN-*α* boosted and i.n Ag85B immunized mice produced a higher amount of IL12p70 (1061 ± 0 pg/ml) compared with BCG-primed and PBS-boosted mice (IL12p70, 899 ± 347 pg/ml), it did not reach statistical difference (Figure 1(b), dark gray).

On the other hand, splenocytes from BCG vaccinated/i.m. IFN-*α* boosted mice and i.n. immunized with Ag85B elicited a higher amount of TNF-*α* (27267 ± 717 pg/ml) (*P* < 0.05) compared with BCG-vaccinated and PBS boosted mice (TNF-*α*, 2767 ± 0 pg/ml) (*P* < 0.05) (Figure 1(c)). Lymph node cells from BCG-vaccinated/i.m. IFN-alpha boosted mice, i.n challenged with Ag85B, did not produce a statistical significance for TNF-*α* (Figure 1(c)). Lung cells from BCG vaccinated/i.m. IFN-*α* boosted mice (Figure 1(c)) produced a substantially higher amount of TNF-*α* (43100 ± 2337) (Figure 1(c)) (*P* < 0.05) compared to nonvaccinated control mice (TNF-*α*, 2433 ± 269 pg/ml) and BCG-vaccinated, nonboosted mice (TNF-*α*, 3433 ± 179 pg/ml) (*P* < 0.05) (Figure 1(c)).

### 3.2. Intramuscular IFN-Alpha Boosting of BCG Immunity Protects Adult Mice against* M. tuberculosis* Infection

Since the IFN-alpha boost Th1 type BCG-inducing immunity (Figure 1(b)), either at the mucosal or systemic level, a prime boost protocol was designed (Figure 2(a)) with the aim of determining protective effect of the IFN-alpha boosting in the mouse model of tuberculosis (Figure 2(a)). At certain periods of time, primed mice were intramuscularly (i.m.) boosted with IFN-alpha and* M. tuberculosis* challenged (Figure 2(a)). The reduction of CFUs (measure of protection) was assessed in spleen and lung eight weeks after challenge. BCG-vaccinated adult mice boosted with IFN-*α* by the i.m. route showed a very small drop in the bacterial load in lungs by 0.3 logs, compared to BCG-vaccinated mice, nonboosted, and by 1.4 logs with respect to control PBS immunized mice (Table 1). In the spleen, BCG-vaccinated/i.m IFN-alpha boosted mice had a 0.9 log reduction in bacterial load (Table 1), compared with BCG-vaccinated mice, and 1.9 logs less bacteria than control mice (Table 1). IFN-alpha i.m. administration alone did not induce any protection, strengthening the fact that type I IFNs (*α*/*β*), either by intranasal or parenteral route, can exacerbate* MTb *infection [17]. However, under the vaccination settings used in this study, like BCG priming and IFN-alpha boosting, we found that IFN-alpha induced a protective effect against* MTb* aerosol infection. The observed systemic protection correlated with a specific HBHA Th1-type cytokine (IFN-*γ*, IL-12, TNF-*α*, and IL-17,) production (Figures 2(b) and 2(c), gray and dark gray bars) elicited by splenocytes and lymph node cells.

### 3.3. Interferon Alpha Associated with BCG Vaccine Pretreatment of Epithelial Cells and Derived Macrophages from Human PBMC Drives Th1 Inducing Production and Restricting Bacterial Growth

**Figure 3:**
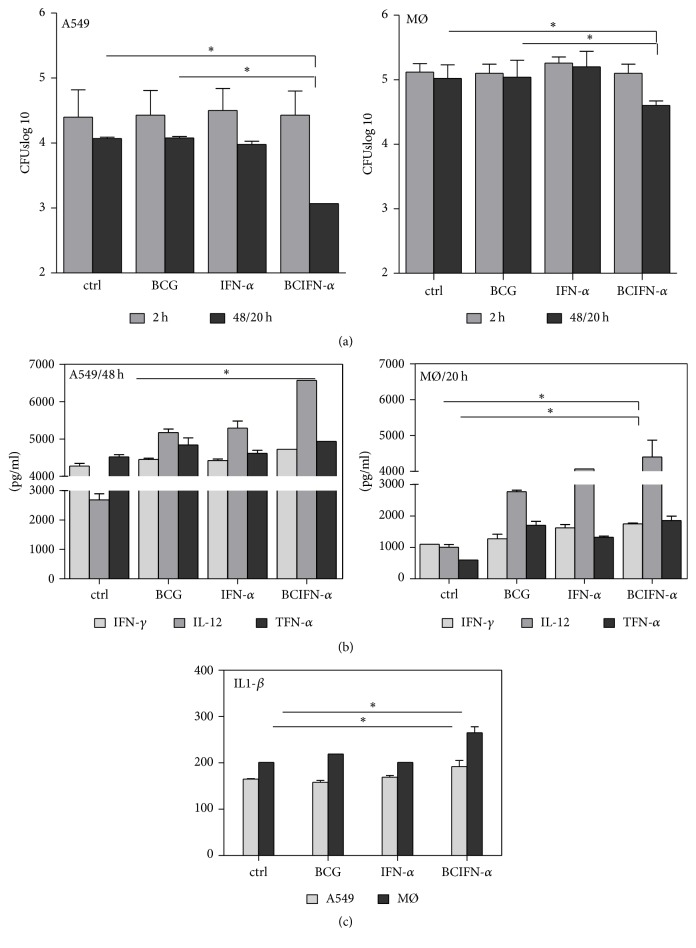
(a) Interferon alpha associated with BCG vaccine pretreatment of epithelial cells and derived macrophages from human PBMC drives Th1 type cytokines production and restricted bacterial growth. Monolayers of pneumocytes type II (A549 cell line) were pretreated with IFN-alpha (500 UI) and BCG (MOI of 10 : 1). After 24 h, these cells were* M. tuberculosis* infected with a MOI of 1 : 10. A significant reduction of bacterial load (by 1 log) with respect to human bronchial epithelial cells/PBS or BCG treated (*P* < 0.05) and infected cells. In derived macrophages from human PBMC, at 20 h after infection, a 0.4 log reduction was observed. (b-c) In each case IFN-*γ*, IL-12, TNF-*α*, and IL1-*β* were determined in the supernatant by immunoenzymatic assay (ELISA).

To strengthen IFN-alpha booster effect on BCG vaccine in order to reduce bacterial load in the mouse model of tuberculosis, monolayers of pneumocytes type II (A549 cell line) were pretreated with IFN-alpha (500 UI) and BCG (MOI of 10 : 1). After 24 h, these cells were* M. tuberculosis* infected with a MOI of 1 : 10. A significant reduction of bacterial load (by 1 log) with respect to human bronchial epithelial cells/PBS or BCG treated (*P* < 0.05) (Figure 3(a)) and infected cells. In derived macrophages from human PBMC, at 20 h after infection, a 0.4 log reduction was observed (Figure 3(a)). In each case, IFN-*γ*, IL-12, TNF-*α*, and IL1-*β* were determined in the supernatant by ELISA (Figures 3(b) and 3(c)). It is noteworthy that the observed bacterial load reduction was correlated also with an increase in these cytokines. Therefore, these data not only agree with those reported by Giacomini et al., 2009, about IL-12 inducing capability by IFN-beta from MoDC BCG or* MTb* infection, but also strongly suggest that, under appropriate settings, IFN-alpha can boost BCG-inducing immunity against* M. tuberculosis* growth.

## 4. Discussion

In the present work, we are reporting that under specific settings IFN-alpha boosting of BCG vaccine can drive the development of specific antimycobacterial cellular immune responses and restrict* M. tuberculosis* growth either in vitro and in vivo.

Several studies have established the role of IFN-I as natural immune adjuvants for commercial vaccines [17–19]. The role of type I IFNs against mycobacterial infections is controversial and depending of the experimental settings. Thus, it has been reported that type I IFNs (beta) can exacerbate the infection; presumably by hampering the development of the Th1 cellular immune responses [17]. Giacomini et al. [27] have shown that IFN-beta endowed BCG-infected DC with the capacity to polarize Th1 cellular immune response, through IL-12 production, suggesting that IFN-beta could be a possible candidate vaccine [27]. To note is the fact that therapy of tuberculosis could be based on innate response, since it has been found that in tuberculosis patients there is an imbalance of the eicosanoid synthesis [20]. Therefore, promotion of this synthesis with some drugs can limit IFNs type I and restrict* MTb* growth [20]. In a different experimental settings, we have reported that successive IFN-alpha administration on BCG-vaccinated mice protected it against* M. lepraemurium* [21], accompanied by a difference in nitric oxide synthase induction [21], while no effects in the Th1/Th2 cytokine pattern were found [21]. Thereafter, and upon this result, we hypothesized that type I IFNs should be administered in association with BCG vaccine, in order to exert an adjuvant effect [28, 29]. The data obtained in this work support this, since interferon alpha boosting of BCG vaccine drives also the development of a specific antimycobacterial Th1-type inducing immunity either in the mouse model of tuberculosis (Figures 2(b) and 2(c); Table 1) or in human celll lines (Figures 3(b) and 3(c)), leading to a restriction of the* MTb* growth (Figure 3(a)). The data are not contradictory to those reported elsewhere [17], since the group of mice treated only with IFN-alpha showed a higher bacterial load while the group of mice vaccinated with BCG and IFN-alpha did not. We reason that the combined action of IFN-*α* and BCG vaccine could be playing an important role in maintaining a more activated state to maintain killing macrophages properties and T cell alive [1, 3, 22]. In addition, recent data from the literature show that the use of BCG and IFN-alpha increases the proinflammatory cytokine production like TNF-alpha [35, 36In contrast, IFN-alpha induction has been related to an increase in IL-10 production, which interferes with IFN-*γ* induction, essential for macrophage activation. Therefore, the use of IFN-alpha alone hampers the control of bacterial growth. Current efforts are being made in order to dissect gene expression profiles of IFN-alpha associated with BCG vaccine in vitro to identify those genes that can serve as biomarker of either host innate or adaptive immune response (iNOS, eicosanoid production, or antimicrobial peptides), or other components of the IFNs signalization that might be involved in the resistance to* MTb*. The understanding and integration of the different actors of both types of immune responses and for some others are still unidentified; we think that they are key for the development of candidate vaccine adjuvants against tuberculosis.

In summary, we are reporting a novel protocol that is based on IFN-alpha alpha boosting of BCG-inducing immunity that might be explored as a potential alternative immunotherapy against pathogenic mycobacteria infections.

## Figures and Tables

**Table 1:** A prime-boost protocol based on BCG-priming and IFN-alpha protects adult mice from *M. tuberculosis* aerosol challenge.

Vaccine	Lung CFUs^a^	Lung protection		Spleen CFUs^a^	Spleen protection	
		control versus BCG			control versus BCG	
Control	5.1 ± 1.27			4.7 ± 0.71		
BCG	4.0 ± 0.99	1.1^*∗*^		3.7 ± 0.78	1.0	
IFN-*α*	4.8 ± 0.64	0.3		4.4 ± 0.14	0.3^*∗*^	
BCG-IFN-*α*	3.7 ± 0.37	1.4^*∗*^	0.3^*∗∗*^	2.8 ± 0.67	1.9^*∗*^	0.9^*∗∗*^

^a^Data are represented as mean ± standard deviations of the log_10_ –transformed bacterial (CFUs) of *M. tuberculosis* per organ. Aerosol challenge. Protection is calculated with respect to control of vaccination (BCG) and with respect to PBS-immunized newborn mice (control). The data reported are ^*∗*^significant at *P* < 0.05 with respect to control mice and ^*∗∗*^significant at *P* < 0.05 with respect to BCG control of vaccination with no boost. One representative of a total of two independent experiments is shown.
